# A comparative study of modified performance based plastic design methods for seismic design of RC frames: improvement over existing methods

**DOI:** 10.1038/s41598-026-45796-4

**Published:** 2026-04-04

**Authors:** Rohit Vyas, Rajneesh Sharma, Venkata Vamsi Emani, Ahmad Batah, Anoop I. Shirkol, Kaushik Gondaliya, Abdullah Ansari, Ayed E. Alluqmani, Pranjal Mandhaniya, Varsha Rani, Bush Rc

**Affiliations:** 1 Department of Civil Engineering, Malaviya National Institute of Technology Jaipur, Rajasthan, 302017 India; 2 Poornima University, Jaipur, Rajasthan 302017 India; 3 Engineering College Jhalawar, (Dept. Of Civil Engineering), Jhalawar, Rajasthan India; 4 Aguirre Project Resources LLC, Texas, 75068 USA; 5 Department of Infrastructure Engineering, The University of Melbourne, Parkville, VIC 3052 Australia; 6 Department of Civil Engineering, Graduate School of Engineering and Technology Ahmedabad, Ahmedabad, 382424 India; 7 Earthquake Monitoring Center, Sultan Qaboos University, PC: 123 Al Khoudh, Muscat, Oman; 8 Department of Civil Engineering, Faculty of Engineering, Islamic University of Madinah, Al-Madinah Al-Munawarah, Medina, Saudi Arabia; 9 Department of Mechanical and Industrial Engineering, Norwegian University of Science and Technology Trondheim, Trondheim, Norway; 10 Government Polytechnic, Purnea, Bihar 854303 India

**Keywords:** Energy-based seismic design, Resilient structural design, Modified performance based plastic design, Post yield stiffness, Fragility analysis, Engineering, Mathematics and computing

## Abstract

This study introduces a Modified Performance-Based Plastic Design (MPBPD) method to enhance the seismic performance of reinforced concrete special moment-resisting frames. Conventional Force-Based Design (FBD) and Performance-Based Plastic Design (PBPD) approaches often underestimate seismic demands due to the neglect of post-yield stiffness. The proposed MPBPD framework explicitly incorporates the post-yield stiffness ratio (${Rt}_{o}$) into the energy modification factor (${\Upsilon }_{o})$, enabling a more realistic representation of inelastic structural response. A comparative investigation is carried out on 6- and 12-storey RC frames designed using FBD, PBPD, and MPBPD through nonlinear static pushover analysis, nonlinear time history analysis, and incremental dynamic analysis. The results show that MPBPD significantly enhances structural capacity, achieving up to 1.77 and 1.47 times higher base shear than FBD for 6- and 12-storey frames, respectively, while maintaining performance points within the Collapse Prevention limit under maximum considered earthquake conditions. Incremental dynamic analysis indicates that MPBPD reduces the median maximum inter-storey drift by approximately 19.5% and substantially lowers the number of ground motions exceeding the 2% drift threshold compared to FBD and PBPD. Probabilistic fragility analysis further demonstrates improved seismic reliability, with Immediate Occupancy and Collapse Prevention states attained at median spectral accelerations of 1.46 g and 5.11 g, respectively. Overall, the MPBPD method provides more uniform drift distribution, reduced seismic vulnerability, and enhanced reliability, highlighting the importance of explicitly accounting for post-yield stiffness in performance-based seismic design of RC frames.

**Supplementary Information:**

The online version contains supplementary material available at 10.1038/s41598-026-45796-4.

## Introduction

Reinforced concrete special moment-resisting frames (RC-SMRFs) continue to be one of the most common lateral force resisting systems in seismic regions because they can develop stable inelastic behaviour and provide significant energy dissipation through ductile flexural mechanisms. In Indian practice, as in many other regions, RC frames are still primarily designed using force-based procedures, where inelastic response is represented indirectly through response reduction factors and code-specified detailing rules^1^. This approach has the advantage of being simple and familiar, but it does not explicitly connect design choices to expected damage states, residual deformation, or story-wise drift distribution under strong earthquakes. As a result, two designs that appear similar in terms of code base shear can behave quite differently in nonlinear response, especially when stiffness degradation, P–Δ effects, and strength deterioration begin to govern response. This mismatch between code-level force design and actual nonlinear performance is one of the main reasons why performance-based seismic design frameworks have been promoted over the last few decades, with the aim of linking design parameters to explicit drift limits and damage states, and evaluating performance under realistic nonlinear demands^2–4^.

Within this broader shift, Performance-Based Plastic Design (PBPD) has gained attention because it offers a direct and transparent design route: a target drift and a desired plastic mechanism are selected upfront, and the required strength is obtained using an energy balance concept together with plastic design principles^5^. Compared to conventional force design, PBPD is attractive because it gives the designer a clearer handle on (i) the intended yield mechanism (typically strong-column weak-beam), and (ii) the drift level that the structure is expected to reach at the design hazard level^6^. PBPD has been applied to steel and RC frames and has often been reported to give improved drift control and more mechanism-consistent designs when compared with traditional approaches^7^. However, recent literature also shows that the reliability of PBPD predictions depends strongly on the assumptions used to represent post-elastic behavior^8,9^. Classical PBPD formulations generally idealize the equivalent SDOF response as elastic–perfectly plastic, while real RC frames exhibit stiffness degradation, cracking-induced softening, pinching in hysteresis loops, and possible strength deterioration^10^. These features influence how and where drift accumulates along the height, and they also affect residual drift and stability during strong shaking. Experimental and numerical observations reported by Liao and Goel (2012) made it clear that RC PBPD designs can display pinched hysteresis and significant stiffness loss under cyclic loading if the formulation is used without suitable adjustments, and they discussed the need for practical corrections such as modified drift targets and consideration of second-order effects to better align the design assumption with RC behaviour^7^. In the same context, many researchers have also noted that PBPD, in its original form, is typically developed around a single target performance objective; this can be limiting in modern performance-based practice where different hazard levels and multiple performance objectives (serviceability, life safety, collapse prevention) may need to be checked.

Moreover, the selection of lateral force distribution in nonlinear static (pushover) analysis has been widely debated in the literature, particularly for mid- to high-rise structures where higher-mode contributions may significantly influence drift and force distribution along the height^11–13^. For regular low- to mid-rise buildings with dominant first-mode participation, first-mode-based lateral load patterns have generally been shown to provide reasonably accurate estimates of global response parameters such as base shear capacity and overall drift demand. However, as structural height and modal interaction increase, reliance solely on first-mode assumptions may not fully reproduce the inertia force distribution observed in dynamic response. Consequently, advanced procedures such as generalized pushover analysis, consecutive modal pushover (CMP), adaptive pushover analysis, spectrum-based pushover analysis (SPA), extended N2 methods, and multimode pushover analysis have been proposed to better capture higher-mode effects and modal interaction^14,15^.

In this context, several recent studies have focused on improving PBPD-type formulations to better reflect post-yield behavior. A common direction in the literature is to move away from the elastic–perfectly plastic idealization and introduce multi-linear representations that include a post-yield stiffness branch, so that the energy balance and strength estimates better match the actual nonlinear response^16^. For example Zhai et al. (2019), proposed an improved PBPD approach using a trilinear force–displacement model and reported that explicit consideration of post-yield stiffness helps achieve performance objectives more consistently, particularly for taller systems where response is not dominated by a single simple mechanism^17^. Related insights are also available from research on self-centering and rocking systems, where post-yield stiffness characteristics and energy dissipation capacity are known to directly affect strength demand, drift response, and residual deformation. Even though the hysteretic behavior of self-centering systems differs from RC frames, the main message remains relevant: the slope of the post-yield branch and the energy dissipation characteristics materially influence the level of seismic demand predicted by energy-based design frameworks^18,19^. This supports the broader argument that post-yield stiffness is not a minor modelling detail; it is a response-controlling parameter that can shift drift distribution, modify energy demand, and influence collapse margins^20^.

Alongside PBPD developments, displacement-based and pushover-based frameworks have also been refined, and the last few years have seen active discussion on how well simplified nonlinear static procedures can represent dynamic inertia force distributions, especially in taller structures. Direct Displacement-Based Design (DDBD) remains a popular displacement-focused alternative because it designs directly for a target displacement profile and uses an equivalent SDOF representation based on secant stiffness at peak response. In many cases, DDBD has been shown to provide good drift control, but studies have also pointed out that the method may require careful choices of displacement profiles and force distributions to avoid undesirable yielding patterns in taller frames. Shakeri and Dadkhah (2021) reported that standard DDBD may not always meet intended drift targets and yield mechanisms for taller steel moment frames and discussed modifications such as adopting inelastic mode-shape based force distributions^21^. Similarly, the N2 method and its extended variants have continued to be used for performance assessment and design checks, with extensions proposed to better include higher-mode effects and torsion in asymmetric structures. These efforts reflect a wider understanding that the chosen lateral load pattern in pushover analysis—uniform, modal, adaptive, or multi-mode—can influence the estimated ductility demand and the inferred performance point, and therefore should be clearly defined and justified, especially when results are compared across different design approaches^15^. Overall, the recent literature indicates that while simplified methods remain useful for design and comparative research, their assumptions must be stated transparently and their limitations acknowledged in the context of multi-storey behavior.

A consistent theme across recent comparative studies based on nonlinear static analysis, nonlinear time-history analysis, and incremental dynamic analysis is that PBPD provides a strong base framework for drift-targeted, mechanism-controlled design, but further refinement is needed to improve reliability for RC frames where post-yield stiffness degradation and cyclic deterioration influence the story-wise distribution of deformation. At the same time, the comparative literature also suggests that a useful refinement should not discard PBPD’s core strengths—its direct link between energy demand and plastic mechanism control—but should enhance its ability to represent the post-yield branch more realistically. In this background, explicitly incorporating a post-yield stiffness parameter into the PBPD energy formulation becomes a logical step, because it targets a specific source of mismatch between simplified design assumptions and observed nonlinear response. This motivates the development of modified PBPD methodologies for RC-SMRFs aimed at improving drift uniformity, seismic capacity, and reliability under strong ground motions, while remaining practical enough to be adopted within performance-based design workflows.

### Novelty and contribution of the present study

The proposed Modified Performance-Based Plastic Design (MPBPD) framework represents a conceptual refinement of the classical PBPD methodology rather than a numerical or empirical adjustment. While the original PBPD formulation is founded on energy balance principles and has proven effective in achieving drift-controlled and mechanism-based designs, it implicitly assumes an elastic–perfectly plastic response, thereby neglecting the influence of post-yield stiffness on seismic energy demand. This assumption is particularly restrictive for reinforced concrete moment-resisting frames, where stiffness degradation, pinching, and post-yield softening govern drift redistribution and residual deformation under strong ground motions. The present study addresses this limitation at the formulation level by explicitly incorporating the post-yield stiffness ratio into the energy modification factor, thereby reformulating the estimation of inelastic energy demand within the PBPD framework.

Unlike previous PBPD applications, where post-yield behaviour is indirectly accounted for through modified drift limits or empirical correction factors, the proposed MPBPD approach introduces post-yield stiffness as an explicit design parameter derived directly from nonlinear static response. This integration alters the fundamental relationship between displacement ductility, strength reduction, and energy demand, leading to a revised design base shear that more realistically reflects the actual inelastic response of RC frames. Consequently, the proposed modification influences not only the magnitude of design forces but also the resulting member sizing, plastic hinge distribution, and storey-wise drift characteristics, which cannot be achieved through simple parameter tuning within the original PBPD framework.

The novelty of the present work also lies in the systematic validation of the MPBPD formulation through multiple nonlinear performance assessment tools, including nonlinear static pushover analysis, nonlinear time-history analysis, incremental dynamic analysis, and probabilistic fragility assessment. By applying a consistent modelling framework to frames designed using force-based design, PBPD, and MPBPD, the study isolates the influence of post-yield stiffness on seismic response and demonstrates its role in improving drift uniformity, enhancing seismic capacity, and reducing vulnerability across multiple performance levels. The combined formulation-level refinement and comprehensive performance evaluation establish MPBPD as a rational extension of PBPD, aimed at improving the reliability of performance-based seismic design for reinforced concrete moment-resisting frames.

### Modified energy factor with post yield stiffness ratio (${\mathrm{Rt}}_{{\boldsymbol{o}}}$)

The PBPD approach provides a direct design methodology in which the target drift (${\theta }_{max}$) and the intended yield mechanism are defined at the outset. The fundamental expressions of the method, originally formulated evaluates the total inelastic energy demand of a multi-degree-of-freedom (MDOF) system by equating it to the elastic energy requirement of an equivalent single-degree-of-freedom (E-SDOF) system using the principle of virtual work^7^. Within this framework, an energy modification factor ($\Upsilon$) is introduced to represent the ratio of inelastic to elastic energy. This factor, which depends on both the displacement ductility ($\mu$) and the strength reduction coefficient (${R}_{\mu }$), provides a direct means to link seismic force reduction and displacement amplification to the dynamic characteristics of the E-SDOF system. The procedure thereby enables evaluation of displacement ductility for an elastic perfectly plastic (EPP-SDOF) oscillator, while simultaneously guiding member design to meet both strength and drift requirements in the nonlinear range through energy balance principles^7,22^. Sectional sizing is subsequently carried out through plastic design techniques^23^.

It is well recognized that allowing nonlinear behaviour within the PBPD framework results in a reduction of structural stiffness. It is evident from the study conducted by Nakshima et al. (1996), ye et al. (2008) and Zhang et al. (2025) that the positive post yield stiffness in a structural system significantly reduce the displacements and imparts stability which is not directly introduced in existing PBPD method^10,16,24^. To overcome this shortcoming a unique methodology proposed extending the PBPD formulation by explicitly incorporating the post-yield stiffness ratio (${Rt}_{o}$) into the energy balance Eq. ^25,26^. From the force–displacement response of inelastic frames, they derived a modified energy modification factor (${\Upsilon }_{o}$) expressed as a function of $\mu$, ${R}_{\mu }$, and ${Rt}_{o}$. Their findings indicated that the integration of ${Rt}_{o}$ within the MPBPD framework substantially improves the reliability of seismic performance predictions.

The use of energy balance as a basis for limit-state design was first introduced by Housner (1956)^27^. Subsequent refinements incorporated the energy factor ($\Upsilon$) and plastic analysis procedures to compute design base shear and sectional forces. Under conditions of fully elastic behaviour, the maximum input energy is defined as represented in Eq. 1.

1 $$W_{e} = \frac{1}{2}MS_{V}^{2}$$

**Fig. 1:**
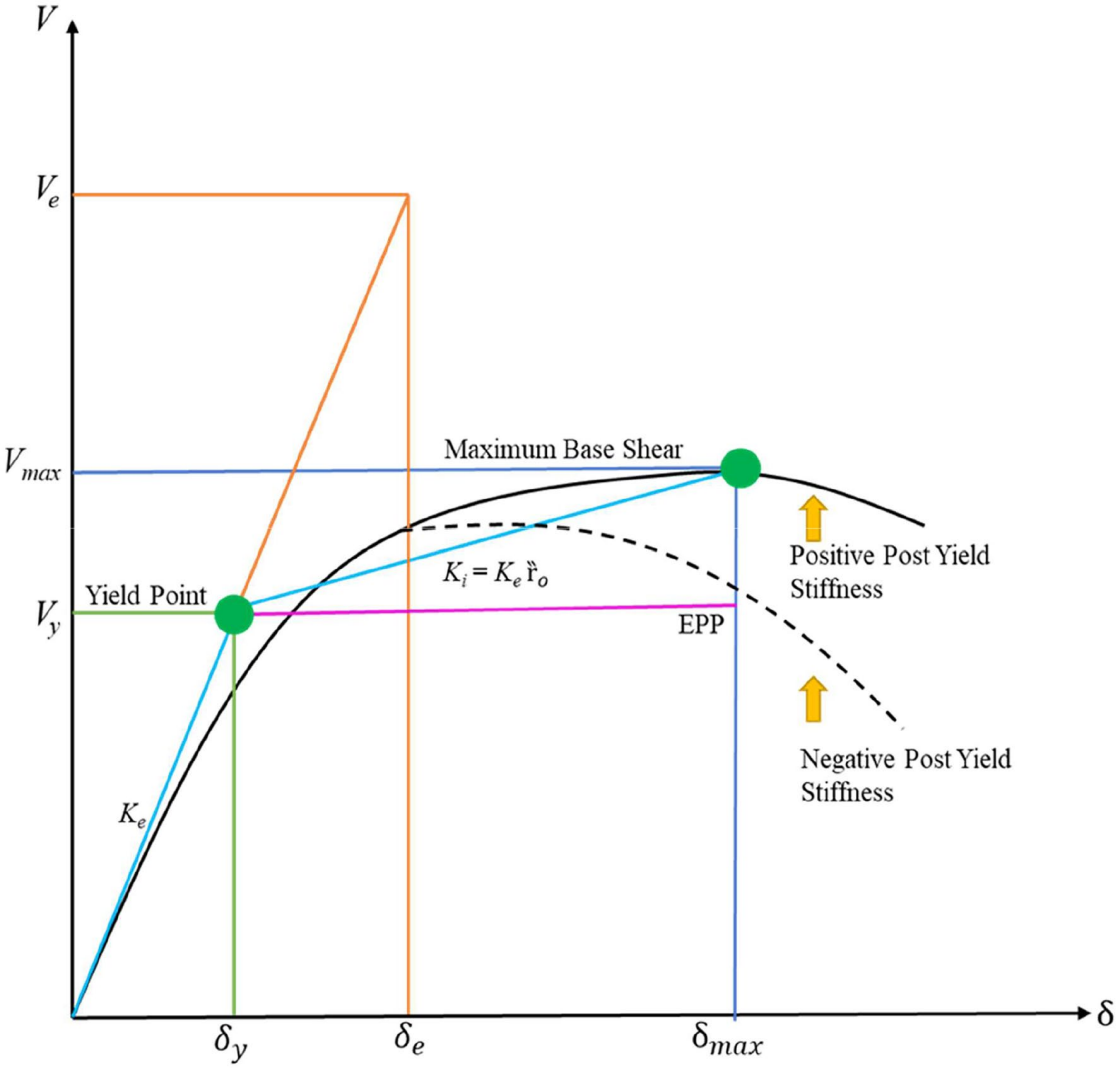
Load Displacement curve with EPP and post yield stiffness ratio (${Rt}_{o}$).

In Eq. 1, ${W}_{e}$ represents the total elastic energy of the system, $M$ is the total mass, and ${S}_{V}$ denotes the spectral velocity. The role of the post-yield stiffness ratio (${Rt}_{o}$) in modifying inelastic behaviour is illustrated through a bilinear force–displacement curve, as shown in Fig. 1. The initial slope,${K}_{e}$ defines the elastic stiffness, with yield displacement ${\delta }_{y}$ and yield strength ${V}_{y}$ marking the transition to the inelastic range. Beyond yielding, two conditions are depicted: an elastic perfectly plastic (EPP) response, characterised by constant strength at ${V}_{y}$ and a response with post-yield stiffness, where the slope of the inelastic branch equals ${K}_{e}.{Rt}_{o}$. The elastic energy of the system is identified as ${W}_{e}$ while the plastic energy is denoted by ${W}_{P}$. The peak base shear,${V}_{max}$ corresponds to displacement ${\delta }_{max}$ whereas the lateral force at ultimate displacement ${(\delta }_{max})$ may be lower than ${V}_{max}$ in the post-peak region. This representation demonstrates how the inclusion of ${Rt}_{o}$ alters the post-yield slope and redistributes elastic and plastic energy, thereby influencing stiffness degradation and energy dissipation capacity as illustrated in Fig. 1. Here ${\delta }_{e}$ and ${\delta }_{y}$ represent the elastic and yield displacements, respectively.

Post-yield behaviour is represented with a bilinear idealization in which the initial (elastic) stiffness ${K}_{e}$ governs the response up to the yield force ${V}_{y}$ at the yield displacement ${\delta }_{y}$ and the post-yield stiffness can be written as ${{K}_{i}=Rt}_{o}{K}_{e}$. The sign and magnitude of ${Rt}_{o}$ determine the slope after yielding, negative values produce softening, ${Rt}_{o}$= 0 corresponds to an elastic-perfectly-plastic response, and positive values give hardening while the specific inelastic mechanism (e.g., P–Δ effects, strength deterioration) controls the extent of that slope. The post yield stiffness of inelastic structural system can also be written as Eq. 2.

2 $${{K}_{i}=Rt}_{o}{K}_{e}=\frac{({V}_{max}-{V}_{y})}{({\updelta }_{max}-{\updelta }_{y})}$$

The displacement ductility ($\mu$) of a structural system can be written as Eq. 3

3 $$\mu =\frac{{\updelta }_{max}}{{\updelta }_{y}}$$

The ratio of yield shear to the yield displacement represents elastic stiffness (${K}_{e}$) can be written as Eq. 4.

4 $${K}_{e}= \frac{{V}_{y}}{{\updelta }_{y}}$$

From Eq. 3, the maximum displacement can be written as ${\updelta }_{max}$=$\mu .{\updelta }_{y}$. The peak base shear written in Eq. 5 attained along the envelope is then obtained by putting Eq. 3 and Eq. 4 in Eq. 2.

5 $${V}_{max}={V}_{y}+{K}_{i} \left({\updelta }_{max}-{\updelta }_{y}\right)={V}_{y}+{\mathrm{Rt}}_{o}{K}_{e}{\updelta }_{y} \left(\upmu -1\right)={V}_{y}[1+{\mathrm{Rt}}_{o}\left(\upmu -1\right)]$$

This Eq. 5 shows that the peak strength exceeds the yield strength when ${Rt}_{o}>0$ (hardening), equals it when ${Rt}_{o}$= 0 (EPP), and falls below it when ${Rt}_{o}$< 0 (softening). In the post-peak range the lateral force at the ultimate displacement ${\updelta }_{max}$ may be smaller than ${V}_{max}$. The elastic and plastic components of the input energy are denoted by ${W}_{e}$ and ${W}_{P}$, with ${W}_{e}$ associated with the triangular elastic portion up to ${V}_{y}$ and ${W}_{P}$ with the inelastic portion beyond ${\updelta }_{y}$. For a structural system, the modified energy factor ${\Upsilon }_{0}$ is defined as the ratio of the inelastic energy demand (${W}_{i}$) to the corresponding elastic energy ${W}_{e}$ as formulated in Eq. 6^26^.

6 $${\Upsilon }_{0}= \frac{{W}_{i}}{{W}_{e}}= \frac{\left\{{V}_{y}{\updelta }_{y}\right\}+\left\{\left({V}_{max}+{V}_{y}\right)\left({\updelta }_{max}-{\updelta }_{y}\right)\right\}}{{V}_{e}{\updelta }_{e}}$$

Factor (${R}_{\mu }$) which reflects the influence of nonlinear behaviour, is defined as the ratio of the elastic strength demand (${V}_{e}$) to the yield strength (${V}_{y}$) , as expressed in Eq. 7.

7 $${R}_{\mu }= \frac{{V}_{e}}{{V}_{y}}=\frac{{\updelta }_{e}}{{\updelta }_{y}}$$

By substituting the expressions from Eq. 5 and Eq. 7 into Eq. 6, and rewriting in terms of ${V}_{max}$, $\mu {\delta }_{y}$ for ${\updelta }_{max}$ and the factor ${R}_{\mu }$ the final formulation of the modified energy factor ${\Upsilon }_{0}$ is derived, as presented in Eq. 8^26^.

8 $${\Upsilon }_{0}= \frac{{Rt}_{o}{\mu }^{2}-\left({Rt}_{o}-1\right)\left(2\mu -1\right)}{{R}_{\mu }^{2}}$$

**Fig. 2:**
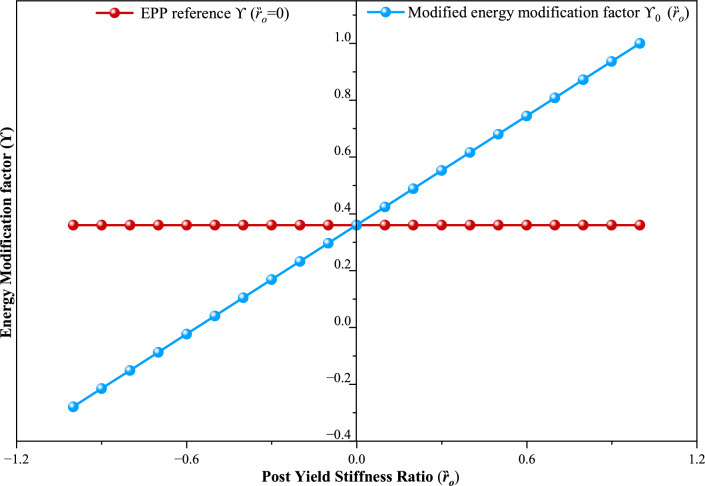
Variation in energy modification factor with variable post yield stiffness ratio.

It is represents by Fig. 2 that the variation in energy modification facto ${\Upsilon }_{0}$ with post-yield stiffness ratio ${(Rt}_{o}$) calculated using Eq. 8. In the Elastic Perfectly Plastic (EPP) case (${Rt}_{o}$=0), Eq. 8 reduces to $(2 \mu -1)/{R}_{\mu }^{2}$ which corresponds to the original PBPD formulation. For softening systems ${(Rt}_{o}<0$)$, {\Upsilon }_{0}$ increases, indicating higher effective energy demand, whereas for hardening systems ${(Rt}_{o}>0$)$, {\Upsilon }_{0}$ decreases, reflecting improved post-yield resistance. The red dashed line denotes the EPP reference value, and the blue curve illustrates how deviations in ${Rt}_{o}$ alter the energy balance. Parametric evaluations further show that ${\Upsilon }_{0}$ increases with larger $\mu$ and decreases with higher ${R}_{\mu }$, underscoring the critical role of both parameters in MPBPD formulations. This refined representation enhances the effectiveness of PBPD by explicitly incorporating post-yield stiffness effects, forming the basis of the MPBPD method.

### Structural modelling and simulations

**Fig. 3:**
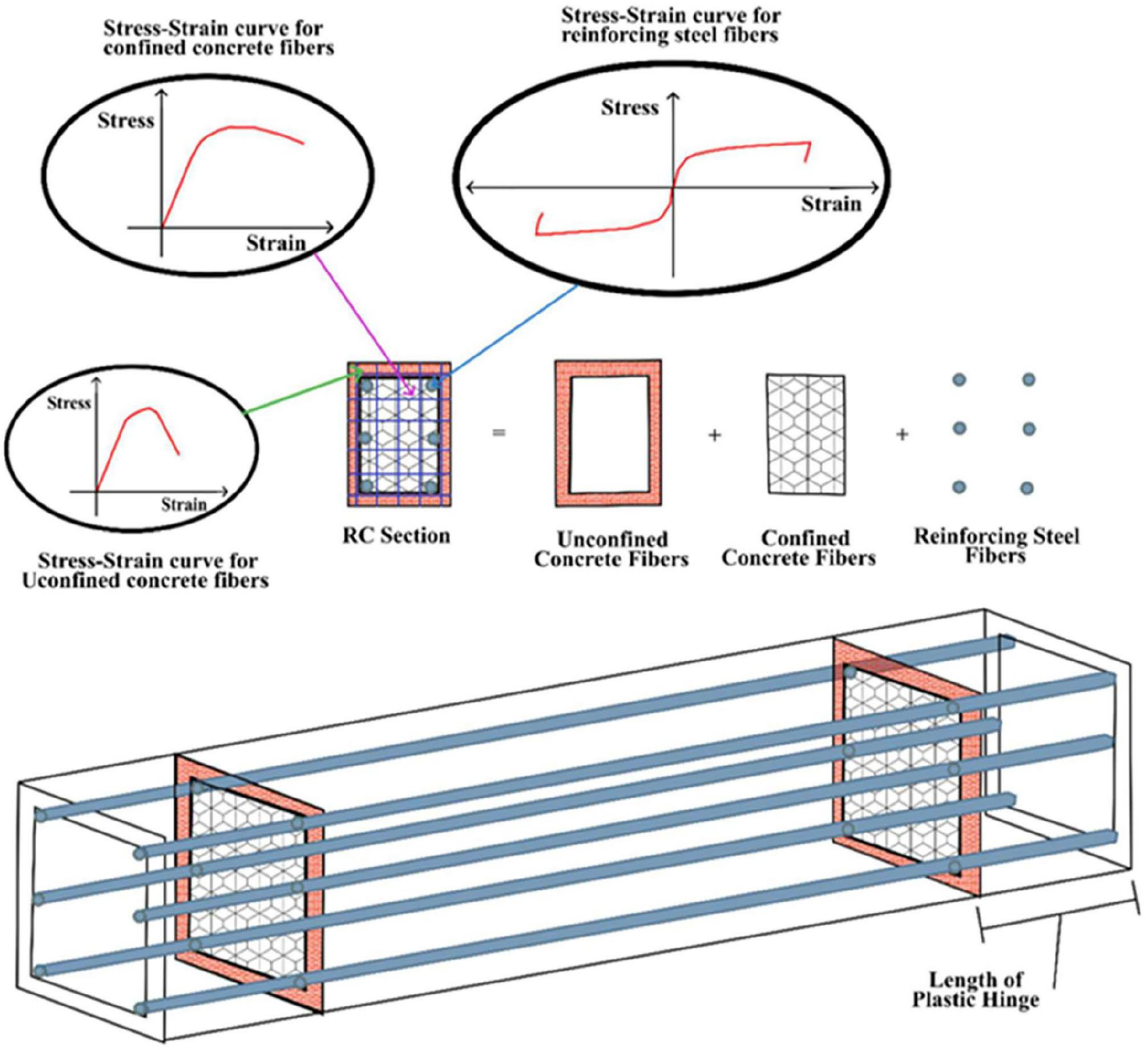
Fibre Modelling Methodology for design members^30^.

The fibre modelling technique was adopted to develop the nonlinear numerical model of the specified frames in ETABS. This approach relies on the stress–strain characteristics of the constituent materials, providing a realistic representation of inelastic structural response. Mander’s model was employed to describe the nonlinear behaviour of confined and unconfined concrete, whereas Park’s model was used for reinforcing steel^28,29^. Accurate definition of these average material properties is essential to obtain reliable simulation results. For this purpose, the expected values of concrete and steel strengths in the nonlinear range were calculated using Eq. 9 and 10. The expected compressive strength of concrete, ${f}_{c,exp}$ is obtained from Eq. 9 as.

9 $${f}_{c,exp}=0.85\times ({f}_{ck}+1.65\times {\sigma }_{c})$$

where ${f}_{ck}$ represents the characteristic compressive strength of concrete and ${\sigma }_{c}$ denotes the standard deviation of concrete strength. Similarly, the expected yield strength of reinforcing steel, ${f}_{y,exp}$, is defined in Eq. 10.

10 $${f}_{y,exp}=({f}_{y}+1.65\times {\sigma }_{s})$$

where ${f}_{y}$ corresponds to the characteristic yield strength of steel and ${\sigma }_{s}$ is the standard deviation of steel strength. These expressions ensure that the nonlinear material properties incorporated in the model adequately capture the variability in concrete and steel strengths, thereby enhancing the reliability of the seismic performance assessment. Each cross-section of the frame was discretized into 24 fibres, with the corresponding stress–strain relationships assigned to capture material nonlinearity. The fibre-based modelling strategy is illustrated in Fig. 3.

**Fig. 4:**
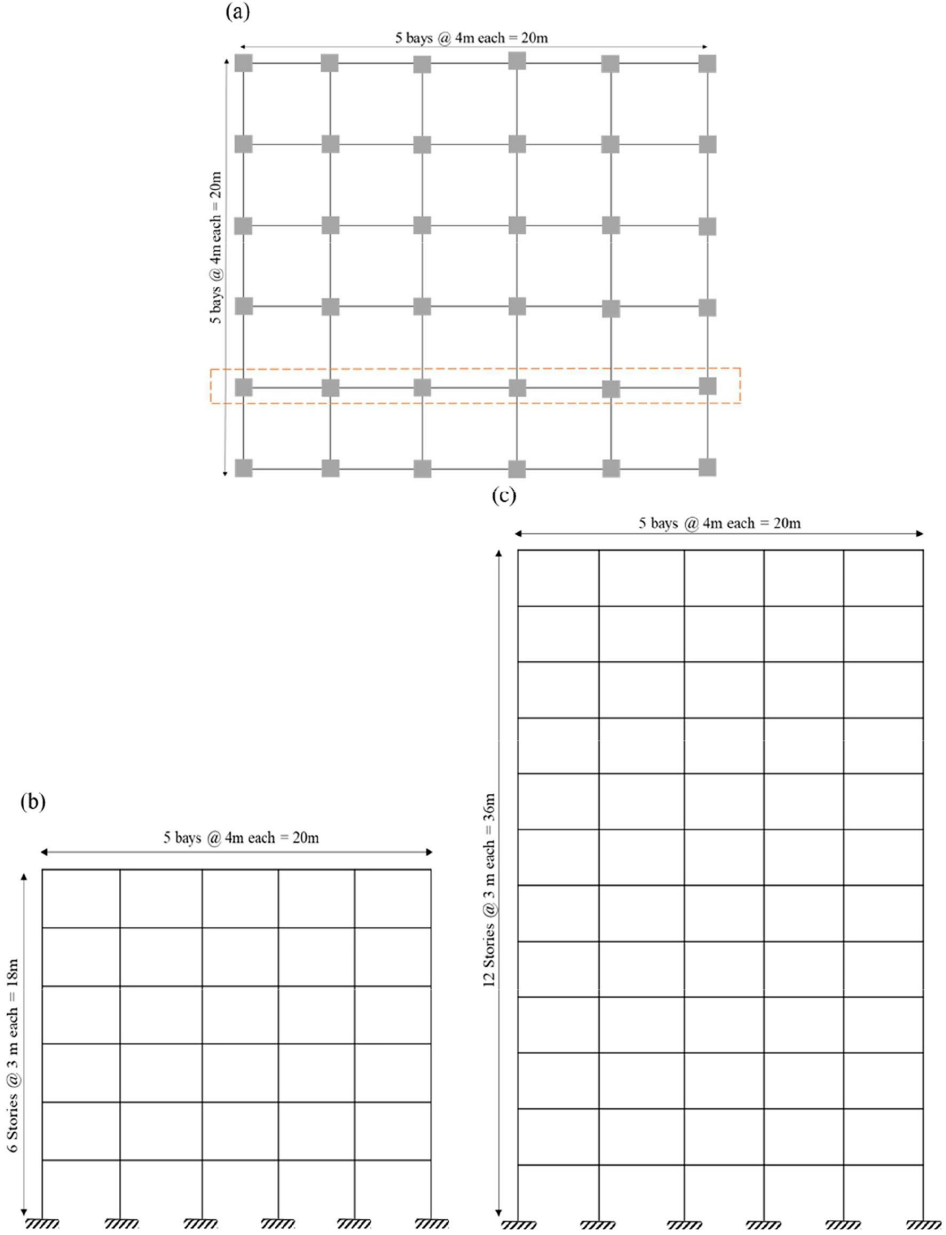
(**a-c**). Structural plan and elevation of 6 and 12-storey study Frame.

To investigate the influence of post-yield stiffness along the height of bare frame structures, a numerical study was carried out on reinforced concrete special moment-resisting frames (RC-SMRFs) of six and twelve stories. Both buildings are regular in plan, consisting of five bays of 5 m in both orthogonal directions, which permitted simplification into two-dimensional analytical models. Each story was modelled with a uniform height of 3 m. The structures were assumed to be located in seismic Zone V, designed with an importance factor of 1.0 and a response reduction factor of 5. The fundamental periods, estimated using the empirical relation ($T= {0.075h}^{0.75}$) were 0.655 s for the six-story frame and 1.102 s for the twelve-story frame. For medium soil conditions (*Type III*), the corresponding spectral acceleration coefficients (${S}_{a}/g$) were obtained as 2.50 and 1.52, respectively. Preliminary member dimensions were assigned in accordance with the provisions given standards of 2000 and 2016^1,31,32^**. **Fig. 4 presents the typical plan and elevation of the two frames. The design load combinations were adopted as per standards of 1987 and 2016^1,33,34^. Concrete of grade M25 was specified, with reinforcement grades Fe500 for longitudinal bars and Fe415 for transverse ties. The total lumped seismic weights of the six- and twelve-story frames were computed as 3481.63 kN and 8594.13 kN, respectively.

### Working principle of performance based plastic design (PBPD)

Unlike conventional force-based design, which indirectly accounts for inelastic behaviour through reduction factors, the PBPD method directly integrates energy balance principles and plastic mechanism concepts into seismic design. Developed to achieve targeted performance under nonlinear demands, PBPD specifies a desired global drift and distributes plastic hinges according to an intended yield mechanism. This approach provides a rational and transparent link between seismic energy input, structural ductility, and required strength capacity. According to ASCE guidelines, the maximum permissible story drift is 1% for masonry-infilled buildings and 2% for other structures^35^. In practice, however, substantial structural and non-structural damage may occur even before the 1% drift limit is reached. Therefore, in the PBPD framework, the target drift (${\theta }_{max}$) is conventionally taken as 0.02 (2%). Similarly yield drift (${\theta }_{y}$) of 0.004 (0.4%) is assumed, from which the total plastic drift (${\theta }_{p}$) is derived^36^. The ductility factor ($\mu$) is defined as the ratio of ${\theta }_{max}$ to ${\theta }_{y}$. The design base shear (${V}_{B}$) and the distributed storey shear (${Q}_{i}$) are evaluated using Eq. 11 to Eq. 15^7^.

11 $${V}_{B}=\left(\frac{- {A}_{h}+ \sqrt{{A}_{h}^{2}+4\Upsilon {S}_{a}^{2}}}{2}\right)W$$

12 $$\Upsilon =\frac{2\mu -1}{{R}_{{\boldsymbol{y}}}^{2}}$$

13 $${A}_{h}= \left(\sum_{i=1}^{n}\left({\beta }_{i}-{\beta }_{i+1}\right){h}_{i}\right){\left(\frac{{w}_{n}{h}_{n}}{{\sum }_{i=1}^{n}{w}_{i}{h}_{i}}\right)}^{0.75{T}^{-0.2}}\left(\frac{{\theta }_{p}8{\pi }^{2}}{{T}^{2}g}\right)$$

14 $${\beta }_{i}={\left(\frac{\sum_{i=1}^{n}{W}_{1}{h}_{i}}{{W}_{n}{h}_{n}}\right)}^{{0.75T}^{-0.2}}$$

15 $${Q}_{i}=\left[\left({\beta }_{i}-{\beta }_{i+1}\right){\left(\frac{{w}_{n}{h}_{n}}{{\sum }_{i=1}^{n}{w}_{i}{h}_{i}}\right)}^{0.75{T}^{-0.2}}\right]{V}_{B}$$

The energy modification factor ($\Upsilon$*)* is expressed as a function of $\mu$ and ${R}_{\mu }$. For systems with a fundamental time period $T>0.55 s$ the factor ${R}_{\mu }$ is taken equal to $\mu$^37^. In the present study, the target drift is further modified using the ${C}_{2}$ factor to account for cyclic strength degradation and pinching in hysteretic loops. The modified drift (${\theta }_{max}$_*_) is obtained by dividing the original target drift (${\theta }_{max}$) by the ${C}_{2}$ factor. This adjustment affects all associated parameters including plastic drift, ductility, ductility reduction factor, and energy modification factor (denoted as ${\theta }_{p}$*, $\mu$*, ${R}_{\mu }$* & $\Upsilon$* respectively^7^. The inelastic spectral acceleration for PBPD is then determined as (${S}_{a}/g$)/ ${R}_{\mu }$. The parameters used in this procedure are summarized in Table 1.

**Table 1:** Parameters used for PBPD study frame.

S. No	Parameters	6 storey frame	12 storey frame
1	$T$(sec)	0.655	1.102
2	${\theta }_{y}$	0.004	0.004
3	${\theta }_{max}$	0.02	0.02
4	$\mu ( {\theta }_{max}/ {\theta }_{y})$	5	5
5	${S}_{a}/g$	0.5	0.303
6	${R}_{\mu }$	5	5
7	$\Upsilon$	0.36	0.36
8	*C*_*2*_ factor	1.24	1.09
9	$\mu$*	4.02	4.60
10	${R}_{\mu }$*	4.02	4.60
11	$\Upsilon$*	0.44	0.39
12	${A}_{h}$	2.95	2.45
13	${V}_{B}/W$ ***%***	3.65	1.44

### Detailed procedure of modified performance based design (MPBPD)

Although PBPD marked a significant advancement in performance-based seismic design, it overlooks the influence of post-yield stiffness, which plays a decisive role in governing residual displacement, story drift concentration, and overall stability. To address this limitation, the MPBPD refines the PBPD framework by explicitly incorporating the post-yield stiffness ratio into the energy balance equation. This modification ensures that the degradation of stiffness after yielding is captured in the design formulation, thereby leading to more realistic and reliable estimation of seismic demands. In this approach, the modified energy modification factor ${\Upsilon }_{0}$ is defined as a function of the $\mu$, ${R}_{\mu }$ and the post-yield stiffness ratio (${Rt}_{o}$). The latter is calculated as the ratio of post-yield stiffness *(*${K}_{i}$*)* to elastic stiffness* (*${K}_{e}$*)* obtained from the capacity curve generated through NLSPA.

**Fig. 5:**
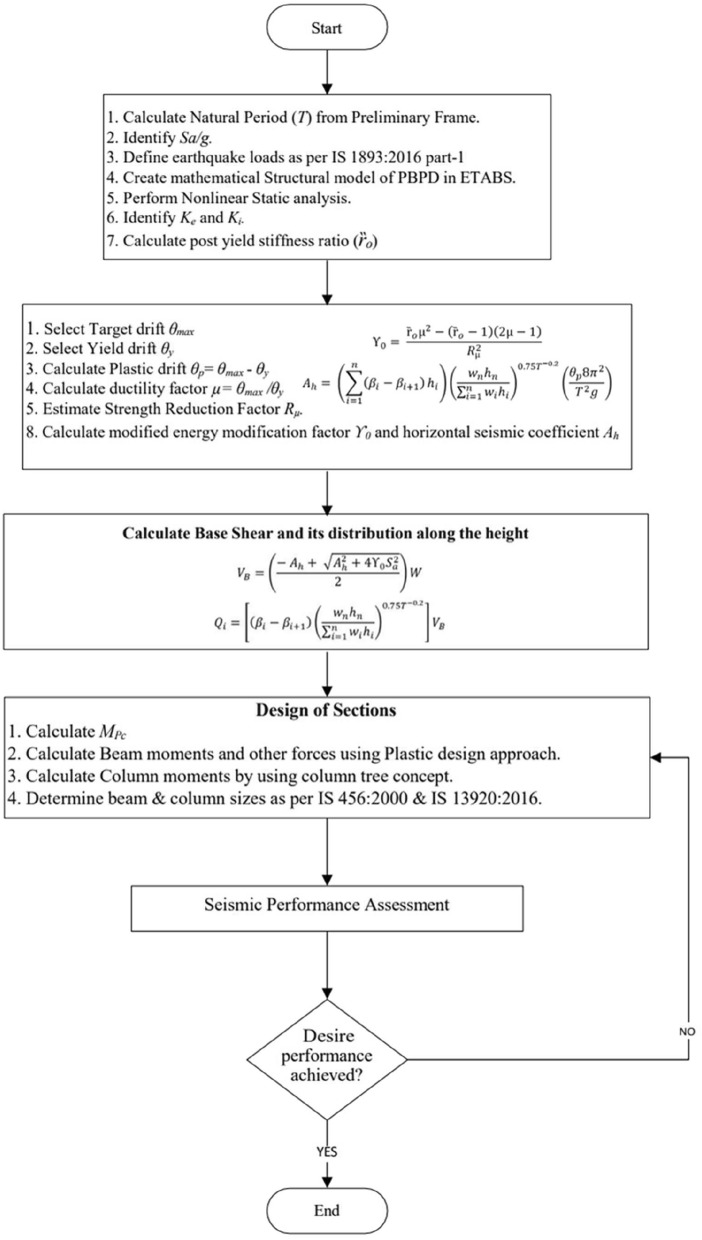
Flow Chart of MPBPD methodology.

The larger design base shear obtained from MPBPD compared with PBPD arises from the explicit incorporation of the post-yield stiffness ratio into the formulation. In the conventional PBPD method, post-yield stiffness is neglected, which may underestimate seismic energy demand and result in comparatively smaller section sizes. In some instances, PBPD may even produce design moments lower than the minimum code requirements, particularly in simplified single-bay two-dimensional frames. In MPBPD, however, the consideration of (${Rt}_{o}$) modifies the energy balance so that the inelastic stiffness is explicitly included, leading to higher estimated design base shear values. This ensures that the sectional dimensions derived in the design process are consistent with the actual post-yield response of the structure. The complete set of parameters used in MPBPD is summarized in Table 2**.**, The overall procedure of the Modified MPBPD, as illustrated in Fig. 5., begins with the evaluation of the natural period of the preliminary frame and identification of the design spectral acceleration^1^. After defining the earthquake load combinations, a PBPD-based model of the frame is developed in ETABS and nonlinear static analysis is carried out to obtain effective stiffness parameters. From the pushover curve, the post-yield stiffness ratio (r₀) is calculated. Using this value, the target drift and yield drift are specified, plastic drift and ductility factor are derived, and the strength reduction factor is estimated. The incorporation of r₀ into the energy balance leads to a modified energy modification factor, from which the design base shear is recalculated and distributed along the building height. Based on these updated demands, beam moments are obtained through the plastic design approach, column moments are derived using the column tree concept, and the final beam and column sizes are proportioned according design codes of year 2000 and 2016^31,32^. As shown in Fig. 5, the designed frame is then subjected to seismic performance assessment; if the desired objectives are not met, section sizes are adjusted and the process is repeated, whereas if the objectives are satisfied, the design is finalized.

**Table 2:** Parameters used for MPBPD study frame.

S. No	Parameters	6 storey frame	12 storey frame
1	$T$(sec)	0.655	1.102
2	${\theta }_{y}$	0.004	0.004
3	${\theta }_{max}$	0.02	0.02
4	$\mu ( {\theta }_{max}/ {\theta }_{y})$	5	5
5	${S}_{a}/g$	0.5	0.30
6	${R}_{\mu }$	5	5
7	${K}_{e}$(kN/m)	5134.06	4798.23
8	${K}_{i}$ (kN/m)	4649.55	4067.52
9	${Rt}_{o}$	0.91	0.85
10	${\Upsilon }_{0}$	0.94	0.90
11	${A}_{h}$	3.91	2.72
12	${V}_{B}/W$_%_	5.92	3.01

Based on the predetermined yield mechanism and the formulations derived from plastic theory, the beam design moments for both PBPD and MPBPD frames were first calculated manually using the plastic design approach^23^. Once the beam mechanism was established, the corresponding column moments were obtained using the column tree concept. In this concept, columns are idealized as continuous vertical elements, analogous to a tree trunk, that transfer the plastic hinge forces developed in the beams through equilibrium conditions at the beam–column joints. This simplification ensures compatibility with the strong-column weak-beam philosophy, which is central to PBPD-based formulations, and allows the column demands to be consistently evaluated without requiring a full nonlinear frame solution at the preliminary design stage. Following the determination of beam and column moments, all members were proportioned using the limit state design methodology^31,32^. The cross-sectional dimensions and reinforcement ratios of beams and columns were selected to ensure both strength and ductility under nonlinear seismic demands. In particular, the PBPD and MPBPD frames were detailed to sustain controlled plastic hinging while avoiding premature brittle failure modes. To capture realistic nonlinear behaviour, Section hinges were derived from M–ϕ analysis using expected material strengths^28,29^. With ${L}_{p} = 0.08L+0.022 {f}_{y}{d}_{b}$ , cord rotations followed θ≈ϕ ${L}_{p}$ and hinges were placed at ${L}_{p}/2$ from the face^38^. Where $L$ is the member length.${ f}_{y}$ is the yield strength of reinforcement, and ${d}_{b}$ is the bar diameter. Beams were modelled with M3 rotational hinges, while columns were assigned PMM interaction hinges. User-defined trilinear backbone curves were adopted, and the IO/LS/CP acceptance rotation limits were defined in accordance with ASCE/SEI 41–17, explicitly accounting for axial load ratio ($\mathrm{n}={\mathrm{P}}_{u} /({\mathrm{A}}_{g}{\mathrm{f}}_{c}\mathrm{E})$, shear demand ratio $({\mathrm{V}}_{y}\mathrm{E}/{\mathrm{V}}_{0}\mathrm{E}<0.30)$ to ensure flexure-controlled behavior, and confinement detailing corresponding to 8 mm transverse ties @150 mm. The exact hinge rotation limits used for beams and columns are summarized in Table 3 for clarity and reproducibility. For consistency, the same hinge property definition procedure was applied to all three design alternatives (FBD, PBPD, and MPBPD), ensuring that differences in nonlinear response arise only from the variation in section sizes and reinforcement produced by the respective design methodologies.

**Table 3:** Hinge rotation limits used for beams and columns.

Component	Axial load ratio (n)	Shear demand ratio	IO (rad)	LS (rad)	CP (rad)	Detailing	Source
RC Beam (250 × 380–430 mm)	Axial effect neglected	< 0.30 (flexure-controlled)	0.005	0.02	0.03	Fe500, M20	ASCE 41–17, Chapter 10
Low Axial (Corner Column)	0.05 – 0.10	< 0.30	0.004	0.02	0.028	8 mm ties @150 mm	ASCE 41–17, Table 10–8 & Commentary
Medium Axial (Edge Column)	0.10 – 0.25	< 0.30	0.003	0.015	0.021	8 mm ties @150 mm	ASCE 41–17
High Axial (Interior Column)	0.25 – 0.45	< 0.30	0.0015	0.006	0.0085	8 mm ties @150 mm	ASCE 41–17

Columns were verified to be flexure-controlled; the shear demand ratio remained below 0.30 for all analysed members. Therefore, flexure-based acceptance criteria as per ASCE/SEI 41–17 were adopted^35^. Column rotation limits were selected as a function of axial load ratio and confinement detailing (8 mm ties @150 mm).

The complete section details, including longitudinal reinforcement percentages for beams and columns in both the 6-storey and 12-storey PBPD and MPBPD frames, are thoroughly explained in *Supplementary Materials*. These details highlight the consistency of the design process across the two methodologies while also reflecting the differences introduced by incorporating post-yield stiffness in the MPBPD procedure.

### Evaluation of structural seismic performance

A reliable evaluation of seismic performance forms the cornerstone of earthquake-resistant design, as it directly governs the safety, functionality, and resilience of structural systems under varying levels of seismic excitation. Understanding how a structure responds not only to service-level earthquakes but also to rare, extreme events is essential for quantifying vulnerability and preventing catastrophic failures. In the present study, a comprehensive performance assessment is carried out for six- and twelve-storey reinforced concrete moment-resisting frames, each designed using three distinct approaches: the conventional FBD, the PBPD, and the proposed MPBPD. The comparative investigation aims to highlight the fundamental differences between these methodologies in predicting and controlling seismic response. Rigorous nonlinear analyses are performed in accordance with established performance assessment guidelines to capture behaviour across multiple seismic demand levels, ranging from frequent to rare earthquakes, as recommended^22^.

### Capacity assessment through pushover analysis

The NLSPA technique, also known as pushover analysis, involves the gradual application of lateral loads to the structural system in order to monitor critical seismic parameters such as the yield point, performance levels, and performance point, thereby enabling an understanding of the collapse progression mechanism^35^. This procedure facilitates a comprehensive evaluation of the global structural response as it approaches ultimate failure, capturing both elastic and inelastic behavioural domains. In the present study, nonlinear static pushover analysis was performed using a first-mode proportional lateral load distribution along the building height. The applied storey forces were assigned in proportion to the product of storey mass and first-mode shape amplitude, thereby approximating the inertia force pattern associated with the fundamental mode of vibration. Since the investigated 6- and 12-storey frames are regular in elevation and exhibit dominant first-mode participation, this loading pattern was considered appropriate for estimating global strength and ductility demand. The same lateral load distribution was consistently adopted for FBD, PBPD, and MPBPD frames to ensure a fair and objective comparison among the three design approaches.

Accordingly, the seismic performance of representative 6-storey and 12-storey RC-SMRF buildings was systematically assessed using these three distinct design philosophies. The FBD frames were initially examined through displacement-controlled pushover analysis to identify fundamental response parameters, including ${K}_{i}$, ${K}_{e}$, and ${Rt}_{o}$. The subsequent evaluation of PBPD and MPBPD frames enabled a direct comparative assessment of their seismic capacity, primarily through base shear–displacement characteristics.

**Fig. 6:**
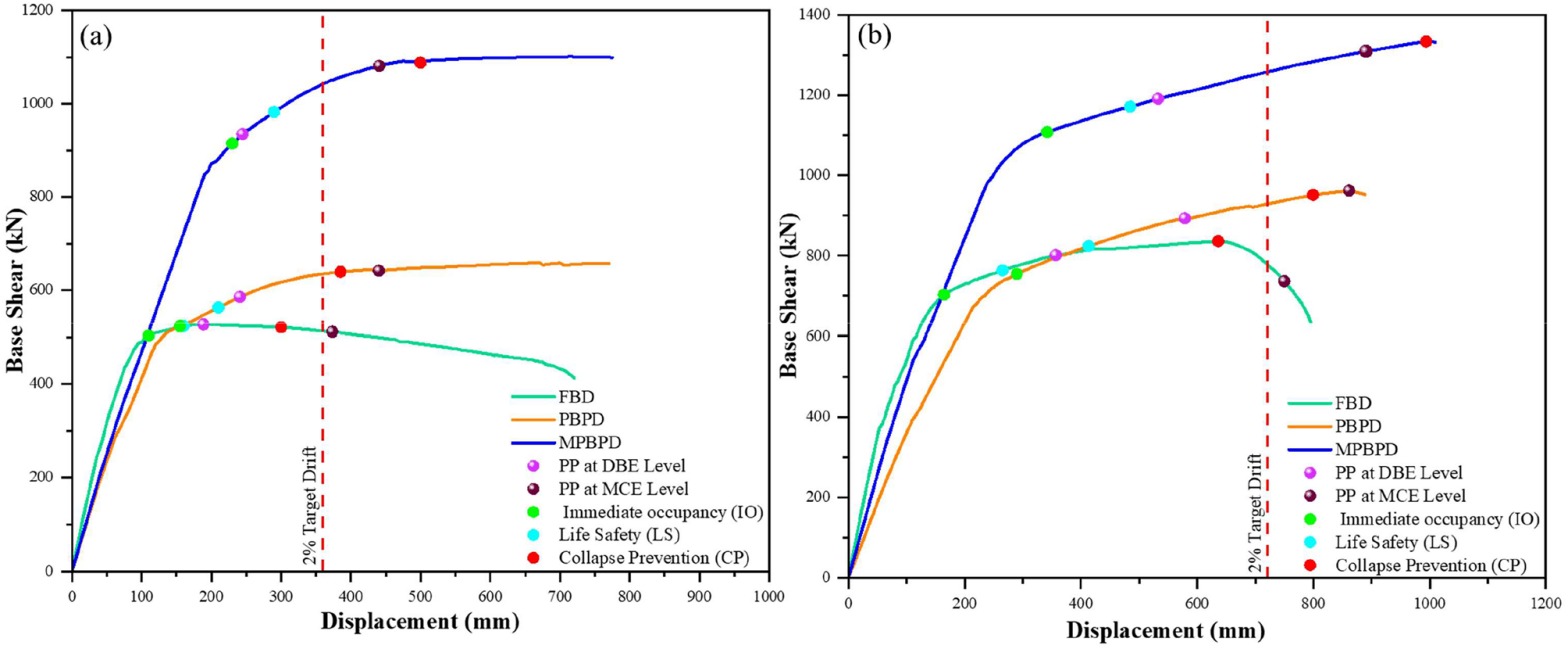
(**a-b**). Pushover curves for the 6 and 12-storey RC-SMRF buildings designed using FBD, PBPD and MPBPD approach.

It is illustrating by Fig. 6** (a-b)** that the pushover curves for the 6-storey and 12-storey frames, respectively. The FBD systems exhibit notable strength and stiffness within the elastic domain but experience marked degradation once their performance point is exceeded. PBPD frames achieve improved performance in the nonlinear regime, reflecting their design philosophy. In contrast, MPBPD frames exhibit enhanced resilience across both linear and nonlinear stages, demonstrating the significance of post-yield stiffness in moderating seismic demand and redistributing structural response.

Quantitatively, the 6-storey PBPD frame achieves 1.51 times the base shear capacity of the FBD frame, whereas the MPBPD frame attains 1.77 times the FBD capacity and 1.18 times the PBPD strength. Similarly, for the 12-storey configuration, the PBPD frame records a strength enhancement of 1.21 times relative to the FBD design, while the MPBPD frame attains 1.47 times the FBD capacity and 1.33 times that of the PBPD frame. Importantly, the Maximum Considered Earthquake (MCE) performance point of MPBPD frames falls within the Collapse Prevention (CP) range for both building heights, indicating their capacity to sustain large inelastic deformations without abrupt strength deterioration. This clearly highlights the superior seismic robustness of MPBPD methodology compared to conventional FBD and PBPD approaches.

### Nonlinear dynamic analysis

In order to obtain a more reliable prediction of structural seismic performance, nonlinear dynamic analysis was carried out on the selected RC-SMRF frames using NLTHA. This approach involves the direct imposition of ground acceleration records at the base of the structures, while the resulting inter-storey drift ratios are recorded for different peak ground acceleration (PGA) levels to develop IDA curves. Since the structural model was developed as a two-dimensional (2D) frame, only the horizontal component of ground motion corresponding to the in-plane direction of the frame was considered in the nonlinear dynamic analyses. The recommendation of use of 18–20 ground motion records ensures a statistically consistent estimation of seismic capacity and collapse potential.

For this purpose, ground motion records were selected to reflect the hazard characteristics associated with the target site. However, given the limited availability of region-specific strong motion data, it is a common practice to supplement the dataset with records from other seismically active regions that possess similar tectonic and spectral properties, even if they originate from different seismic zones. This practice improves the representativeness and variability of the dataset, thereby enhancing the robustness of IDA outcomes.

In line with these recommendations, a total of 22 ground motion records were compiled from the Pacific Earthquake Engineering Research (PEER) strong motion database^39^. The selected dataset encompasses a wide range of magnitudes, epicentral distances, and spectral characteristics to ensure appropriate coverage of structural response variability as described in recent literature^40^. A concise summary of the key details of all chosen ground motion events is presented in Table 4.

**Table 4:** Earthquake record dataset from PEER-NGA Database.

RSN ID	Earthquake event	Magnitude (*M*_*W*_)	Vs30 (m/sec)	Epicentral distance (km)
7	Northwest California-02	6.6	219.312	91.15
9	Borrego	6.5	213.440	56.88
15	Kern County	7.36	385.431	38.42
17	Southern California	6	493.510	73.35
20	Northern California-03	6.5	219.312	26.72
28	Parkfield	6.19	408.933	17.64
36	Borrego Mtn	6.63	213.444	45.12
51	San Fernando	6.61	280.566	55.2
57	San Fernando	6.61	450.284	19.33
121	Friuli_ Italy-01	6.5	496.464	49.13
122	Friuli_ Italy-01	6.5	249.287	33.32
138	Tabas_ Iran	7.35	324.579	24.07
140	Tabas_ Iran	7.35	302.642	89.76
163	Imperial Valley-06	6.53	205.781	23.17
164	Imperial Valley-06	6.53	471.530	15.19
265	Victoria_ Mexico	6.33	471.530	13.8
266	Victoria_ Mexico	6.33	242.052	18.53
280	Trinidad	7.2	311.751	76.06
286	Irpinia_ Italy-01	6.9	496.462	17.51
287	Irpinia_ Italy-01	6.9	356.395	44.62
322	Coalinga-01	6.36	274.734	23.78
323	Coalinga-01	6.36	359.036	55.05

### Normalization and scaling procedure for ground motions

As outlined, all selected ground motion (GM) records are required to be scaled to a prescribed intensity level to ensure consistency in seismic evaluation. Prior to scaling, the records are normalized with respect to peak ground velocity (PGV). This normalization step is essential, as it minimizes the variability introduced by source magnitude, epicentral distance, faulting mechanism, and site effects, thereby yielding a uniform basis for comparison across the dataset. A similar normalization strategy has been adopted in recent literature^41^, where PGV-based scaling is emphasized for achieving spectral compatibility and reducing dispersion in structural response predictions. In the present work, Eq. 16 and Eq. 17 are applied for normalizing the PEER-NGA ground motion dataset.

16 $${NGM}_{i}= \frac{Median ({PGV}_{i})}{{PGV}_{i}}$$

17 $${NGM}_{1,i} = {NGM}_{i x} {GM}_{1,i }\& {NGM}_{2,i} = {NGM}_{i x} {GM}_{2,I}$$

Subsequent to normalization, the records are scaled to the target intensity such that the median spectral acceleration corresponds to the fundamental period ($T$) of the structural system. This procedure ensures that the ensemble of ground motions collectively represents the seismic demand with reduced statistical scatter. The dataset of 22 records compiled from the PEER-NGA repository covers a wide range of PGA and PGV values. Before normalization, PGA values lie in the range of 0.04–0.64g with an average of 0.12g, while PGV values range from 2.32 to 36.07 cm/s with a mean of 13.83 cm/s. After normalization, PGA_max_ values fall between 0.06g and 0.31g, with an adjusted mean of 0.13g, and the normalization factors are observed to vary from 0.10 to 4.0, aligning well with recommendations^42^.

**Fig. 7:**
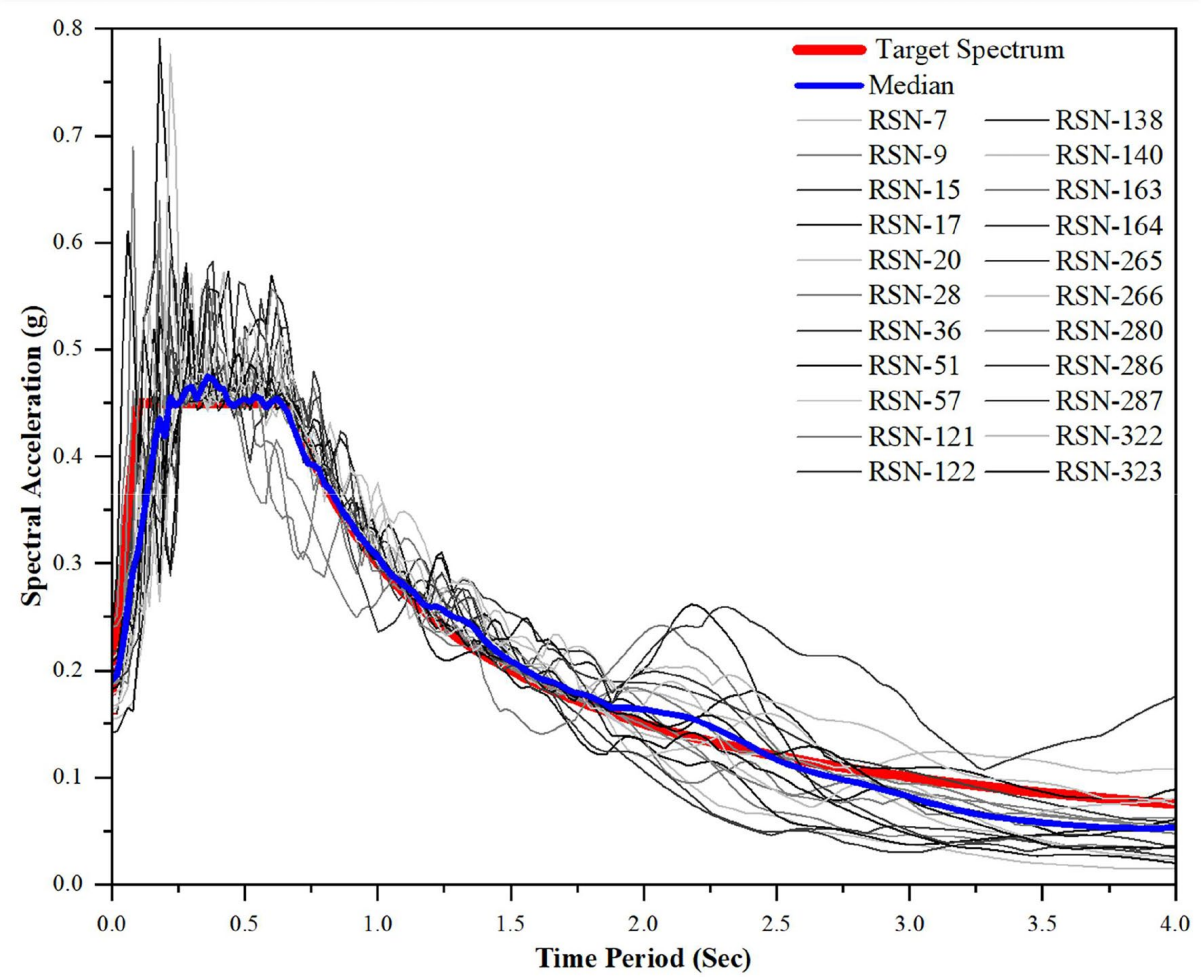
Scaled Response spectra of selected GMs for the Target Spectrum of Seismic Zone-V.

Fig. 7. illustrates the normalized and mean-matched responses of all selected ground motions in comparison with the target response spectrum for Zone V. The normalized and intensity-matched set of records shows a close alignment with the target demand spectrum, thereby confirming that the subsequent NLTHA and IDA analyses capture realistic structural response characteristics while ensuring statistical robustness of the results.

### IDA framework for seismic assessment

**Fig. 8:**
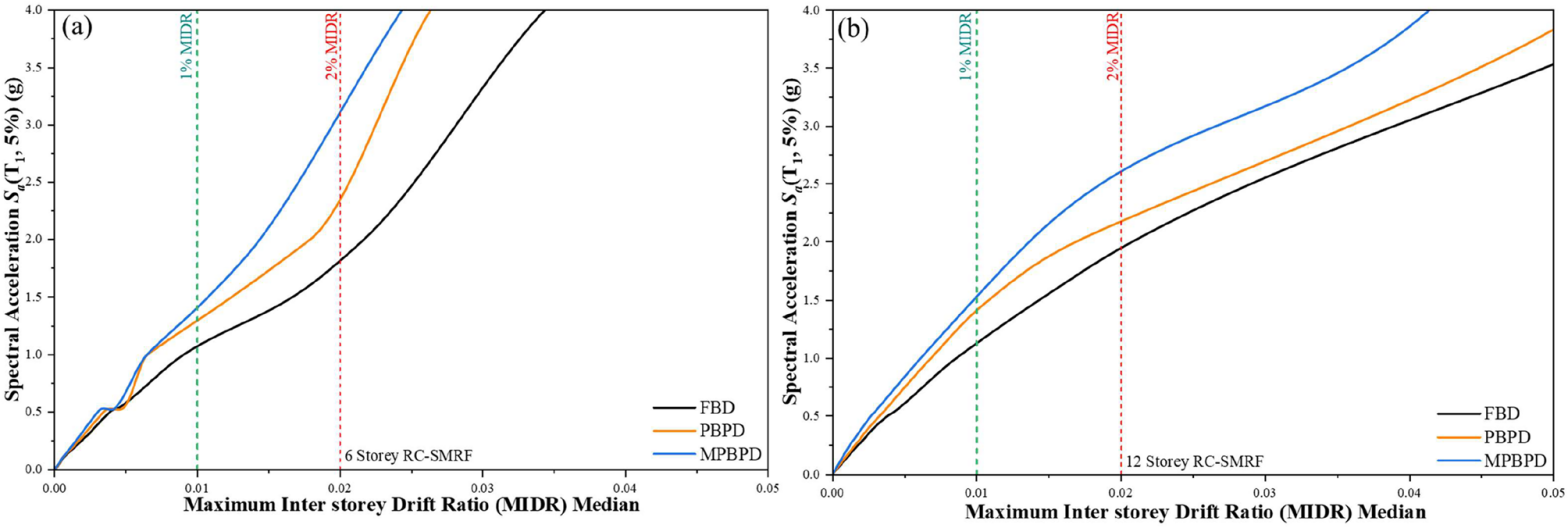
(**a-b**). IDA comparison curve of 6 and 12-storey RC-SMRF for FBD, PBPD and MPBPD approach.

The seismic performance of the frames is evaluated through IDA, where structural models are subjected to ground motion records scaled to increasing intensity levels^43^. Spectral acceleration *Sa* (*T*1,5%), is used as the intensity measure, while the maximum inter-storey drift ratio (MIDR) is taken as the damage measure. In this study, only the median IDA curves are reported to represent the typical response of the 6-storey and 12-storey RC-SMRF buildings designed with FBD, PBPD, and MPBPD methods. Fig. 8(a and b) show these comparisons. For the 6-storey frame Fig. 8(a), the PBPD design shows about 14% lower drift than FBD, while the MPBPD design achieves nearly 22% reduction compared to FBD and around 9% compared to PBPD. At the 1% drift level, MPBPD sustains higher spectral acceleration, showing better transition control. At the 2% drift target, MPBPD again performs best, maintaining higher acceleration capacity than PBPD and FBD.

For the 12-storey frame Fig. 8(b)., a similar trend is observed. PBPD reduces drift by nearly 14% compared to FBD, while MPBPD provides about 29% reduction compared to FBD and 18% compared to PBPD. At both the 1% and 2% drift levels, MPBPD demonstrates the highest resistance, clearly outperforming the other two methods.

**Fig. 9:**
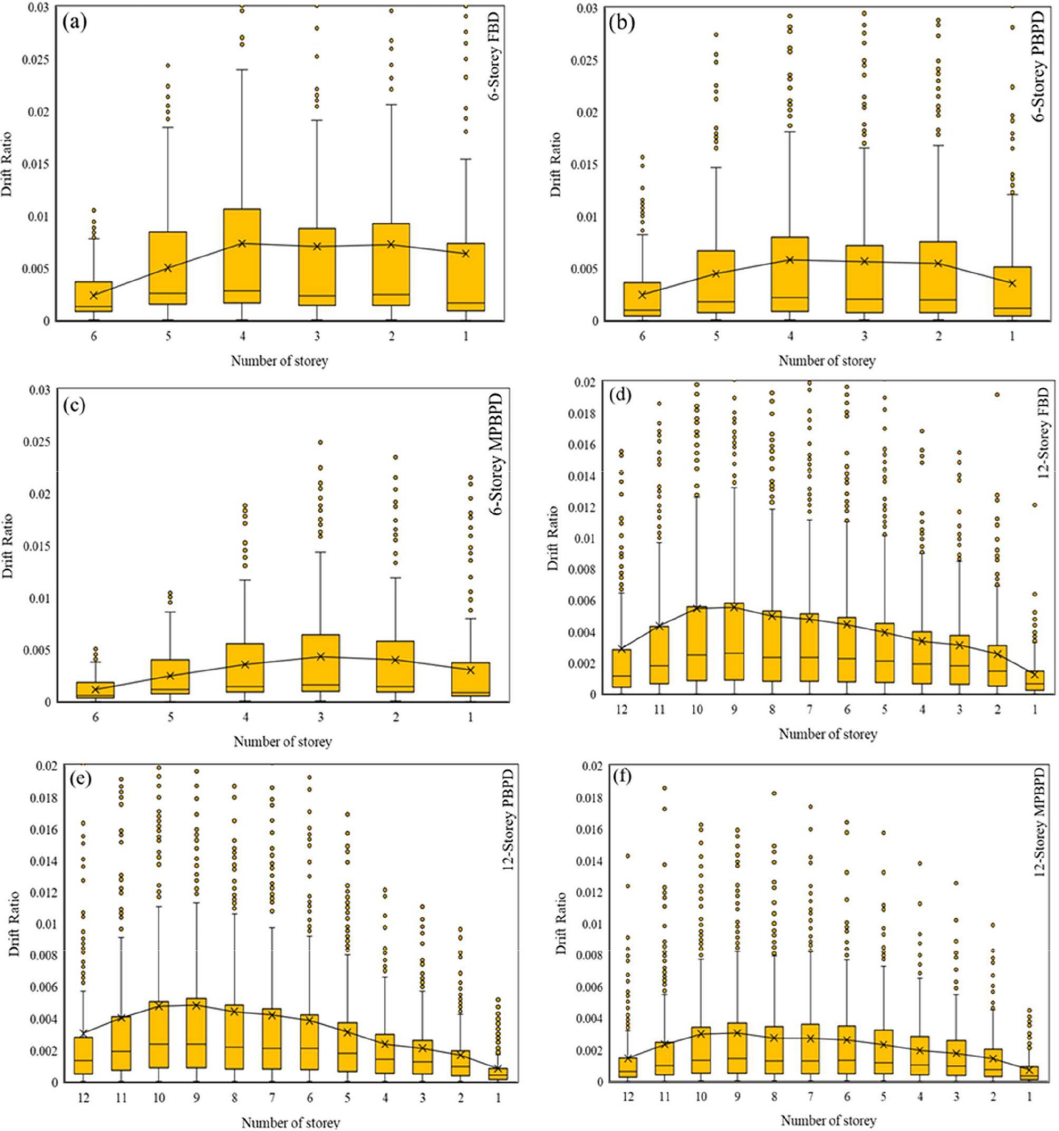
(**a-f**). Box type drift ratio Comparison curve of 6 and 12-storey FBD, PBPD and MPBPD approach.

Overall, PBPD improves the performance relative to FBD, but MPBPD consistently shows the best results. The advantage is especially clear near the 2% MIDR level, which is widely considered a key performance threshold for life-safety, confirming MPBPD’s robustness under strong seismic demands.

storey-level drift distribution was further examined using box plots for both 6-storey and 12-storey frames under FBD, PBPD, and MPBPD design methods Fig. 9(a to f). For the 6-storey frame, the FBD system showed peak drift concentration around the 4th–5th storey, with large variability and several outliers, indicating non-uniform deformation demand. PBPD improved the distribution by reducing extreme values and providing relatively balanced drift across the height, although mid-storey peaks remained evident. In contrast, MPBPD achieved the most uniform drift pattern with reduced variability, demonstrating effective control of inter-storey deformations.

For the 12-storey frame, FBD exhibited maximum drift in the 9th–10th storey, with high scatter and wide variability, while lower and upper storeys experienced significantly smaller values. PBPD provided improvement by lowering drift demands and better distributing deformation across the height, yet mid-storey peaks persisted. MPBPD again demonstrated the most favourable performance, with consistently lower median drift, reduced spread, and a smoother distribution from base to top.

### Fragility analysis

Fragility analysis provides a probabilistic framework to evaluate the likelihood of structural failure under seismic loading, typically expressed in the form of fragility curves^44–47^. These curves relate the probability of exceeding a predefined damage state to increasing levels of seismic intensity. The formulation relies on two fundamental parameters: the median seismic demand threshold, ${\mu }_{k}$, and the logarithmic standard deviation, ${\beta }_{k}$ which together define the central tendency and dispersion of structural capacity, respectively. Previous research has made significant contributions toward the development of fragility functions for reinforced concrete and steel structures, highlighting that the post-yield seismic behaviour of structures is often overlooked by practicing engineers and designers in India. The primary sources of uncertainty in fragility analysis arise from the assumptions in mathematical modelling, the definition of seismic demand spectra, and the selection of damage state thresholds^48–50^. In this context, Engineering Demand Parameters (EDPs), such as maximum displacement or Maximum Inter-storey Drift Ratio (MIDR), are commonly used to quantify structural performance limits. In the present study, fragility curves were developed using IDA data derived from NLTHA. The lognormal cumulative distribution function was employed to represent the probability of exceedance, as expressed in Eq. 18.

18 $$P[DS = DS_{k} |X = \Theta ] = \emptyset \left[ {\frac{1}{{\beta_{k} }}\ln \left( {\frac{\Theta }{{\mu_{k} }}} \right)} \right]$$

Where $P[DS\ge {DS}_{k}|X=\Theta ]$ denotes the probability of a structure reaching or exceeding a specific damage state $D{S}_{k}$ at a given seismic intensity measure $\Theta$. Here, ${\mu }_{k}$ is the median value of seismic intensity corresponding to damage state $D{S}_{k}, {\beta }_{k}$ represents the logarithmic standard deviation that captures uncertainties in structural response, and $\emptyset$ is the standard normal cumulative distribution function. This formulation accounts for uncertainties in structural performance and provides a robust probabilistic basis for quantifying seismic vulnerability.

## Results and discussions

This study examines the seismic behaviour of 6- and 12-storey RC-SMRFs designed using three different approaches: the conventional FBD, the PBPD, and the proposed MPBPD. Comparative evaluation shows that explicitly incorporating post-yield stiffness in the MPBPD framework significantly improves seismic response. Results from NLSPA under MCE conditions reveal that the performance points of FBD and PBPD frames extend beyond the Collapse Prevention (CP) threshold, whereas the MPBPD frame remains within the CP range. This outcome underscores the superior stability and safety margin of the MPBPD method. Moreover, the MPBPD frame exhibits enhanced ductility capacity, reflected in the smoother decline of base shear following peak resistance, indicating improved energy dissipation and structural robustness. Building on these pushover results, IDA using 22 ground motion records further substantiated the advantages of MPBPD. For the 6-storey frame, the 2% drift threshold was exceeded in 14 motions for the FBD design, in 6 cases for the PBPD design, and in only 2 motions for the MPBPD frame. A similar pattern was evident for the 12-storey frame, with exceedances in 12, 5, and 3 cases for FBD, PBPD, and MPBPD, respectively. Drift demand comparisons confirmed that the 6-storey MPBPD frame achieved reductions of 21.89% relative to FBD and 9.14% relative to PBPD, while the 12-storey MPBPD frame demonstrated even larger reductions of 29.38% and 18.18%.

The better performance of MPBPD is mainly because it clearly considers post-yield stiffness in the design calculation. In the conventional FBD method, strength is calculated based only on elastic forces, so it does not properly control where plastic hinges form. Because of this, stiffness reduces quickly after yielding and drift gets concentrated in some middle storeys. PBPD improves this by planning a target yield mechanism, but it still assumes that the structure behaves as elastic–perfectly plastic after yielding, which ignores the beneficial effect of post-yield stiffness. On the other hand, MPBPD includes the post-yield stiffness ratio directly in the energy equation. This increases the required design strength and leads to better and more uniform member sizing along the height. As a result, the structure keeps more stiffness after yielding, drift is more evenly distributed, and inelastic deformation spreads more smoothly. Therefore, MPBPD frames show higher base shear capacity, slower strength reduction after peak load, and better resistance to collapse compared to FBD and PBPD designs.

**Fig. 10:**
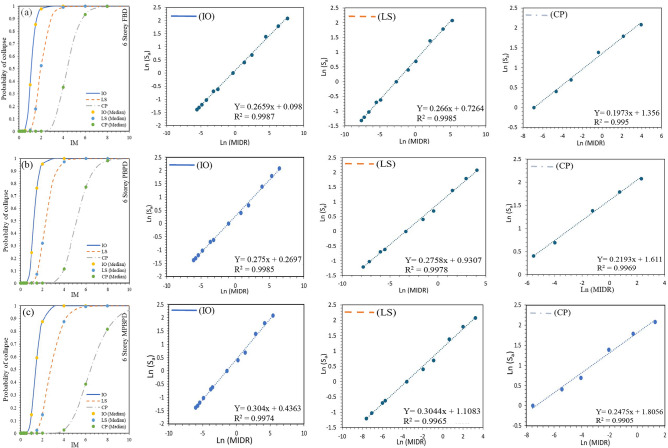
(**a-c**). Traditional IDA curve of 6-storey RC-SMRF for FBD, PBPD and MPBPD approach.

The fragility functions derived for the 6- and 12-storey frames provide a probabilistic measure of seismic performance across different design methodologies. The fragility curve illustrated by Fig. 10**(a-c).** for the 6-storey FBD frame, the probability curves show that the structure reaches Immediate Occupancy (IO) at a median spectral acceleration (${S}_{a}$) of 1.10 g and Collapse Prevention (CP) at 4.34 g, with a standard deviation of 0.293 at IO, reflecting significant variability in seismic demand. The PBPD design improved this behaviour, with median ${S}_{a}$ values of 1.22 g at IO and 5.14 g at CP, and a reduced dispersion of 0.208 at CP, suggesting greater reliability. The MPBPD frame provided the most favourable results, reaching IO at a median ${S}_{a}$ of 1.39 g and CP at 6.44 g, with a comparatively low dispersion of 0.242, thereby indicating its superior robustness under seismic loading.

**Fig. 11:**
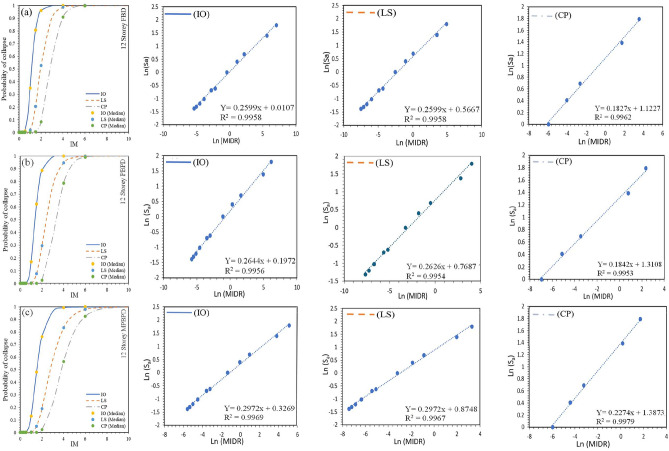
(**a-c**). Traditional IDA curve of 6-storey RC-SMRF for FBD, PBPD and MPBPD approach.

For the 12-storey case, the fragility analysis represented by Fig. 11**(a-c).** indicated that the FBD frame reached IO at 1.13 g and CP at 2.85 g, with a dispersion of 0.255 at CP. The relatively lower Sa capacities and higher variability suggest that the FBD design provides reduced seismic resilience, here defined as the ability to sustain higher intensity demands with lower probability of exceedance and smaller variability across damage states. In contrast, the PBPD frame demonstrated improved resilience, with IO and CP reached at 1.36 g and 3.27 g, respectively, and a reduced dispersion of 0.251. The MPBPD frame again showed the best performance, with IO at 1.53 g and CP at 3.79 g, accompanied by a dispersion of 0.318, thereby confirming its greater capacity to resist seismic forces with dependable reliability.

## Conclusions

Seismic design can be significantly improved by refining conventional plastic design approaches to explicitly capture post-yield behaviour. The MPBPD framework developed in this study addresses the major limitation of the traditional PBPD method, which neglects the influence of post-yield stiffness. By introducing the post-yield stiffness ratio into the energy balance formulation, MPBPD achieves a more accurate representation of inelastic structural response, ensuring higher strength capacity and reduced inter-storey drifts. Its effectiveness has been demonstrated through NLSPA, NLTHA, and IDA. The key findings of the investigation are summarized below:

1. From the capacity curve results, MPBPD frames exhibit 1.77 and 1.47 times greater strength than the 6- and 12-storey FBD frames, and 1.18 and 1.33 times greater strength than the corresponding PBPD frames. These strength improvements clearly confirm the robustness of MPBPD over conventional approaches. In addition, MPBPD frames reach their performance points within the Collapse Prevention (CP) range, allowing them to accommodate larger deformations with a gradual decline in strength and reducing the risk of sudden failure. By contrast, FBD frames show a steep drop in capacity at the performance point, reflecting higher susceptibility to brittle failure under seismic loading. PBPD frames perform better than FBD by improving ductility and reducing drift demands, but they still do not achieve the same level of resilience as MPBPD, which ensures superior strength retention and more reliable post-yield behaviour.
2. The superiority of the MPBPD method is confirmed by IDA results, which show reductions in maximum inter-storey drift of 21.89% and 9.14% for the 6-storey frame, and 29.38% and 18.18% for the 12-storey frame, compared with FBD and PBPD, respectively. Storey-level drift distributions further support these findings: FBD frames exhibited concentrated mid-storey drifts with large variability, PBPD reduced extreme values but retained mid-storey peaks, while MPBPD achieved the most uniform and smoother drift profile with lower variability across the height. These outcomes underline MPBPD’s effectiveness in controlling deformations and minimizing seismic damage.
3. The seismic fragility curves for both the 6-storey and 12-storey frames clearly demonstrate the superior performance of the MPBPD method in mitigating seismic risk compared to the other design approaches. The results indicate that the MPBPD frame reaches the Immediate Occupancy (IO) state at a median spectral acceleration of 1.46 g and the Collapse Prevention (CP) state at 5.11 g, with a minimum dispersion of 0.28. These values reflect the benefits of incorporating post-yield stiffness into the MPBPD formulation, which significantly enhances structural capacity and allows the frames to endure higher levels of seismic demand before entering critical damage states. The relatively lower variability observed in the MPBPD fragility curves further suggests improved consistency and reliability of the response, underscoring the method’s robustness and its potential to deliver resilient performance in seismic-prone regions when compared with both FBD and PBPD.

From a practical design perspective, this study demonstrates that explicitly incorporating post-yield stiffness in seismic design significantly improves drift control, strength reliability, and collapse resistance of RC frames. The MPBPD method provides a more realistic estimation of inelastic demand than conventional force-based and classical PBPD approaches, leading to more uniform drift distribution and reduced risk of soft-storey formation and premature stiffness degradation. These findings indicate that future seismic design codes may benefit from integrating post-yield stiffness effects within energy-based or performance-based design procedures, making MPBPD a practical and implementable approach for enhancing seismic safety, particularly in mid-rise RC buildings. However, the present investigation is limited to numerical analysis of regular 2D frames under medium soil conditions with uniform mass and stiffness distribution. The performance of MPBPD in structures with soft-storey mechanisms, vertical or plan irregularities, torsional effects, or significant mass and strength discontinuities requires further validation. Additional research involving 3D modelling, varied soil conditions, taller buildings, and alternative structural systems such as steel or composite frames is necessary to confirm the broader applicability and robustness of the proposed method.

## Supplementary Information

Supplementary Information.

## Data Availability

The data supporting this study’s findings are available from the corresponding author, Rohit Vyas, upon reasonable request.

## Abbreviations

CSM: Capacity spectrum method
CP: Collapse prevention
EPP: Elastic perfectly plastic
E-SDOF: Elastic single degree of freedom
FBD: Force based design
GM: Ground motion
IDA: Incremental dynamic analysis
IM: Intensity measure
IO: Immediate occupancy
LS: Life safety
MCE: Maximum considered earthquake
MDOF: Multi degree of freedom
MIDR: Maximum inter storey drift ratio
MPBPD: Modified performance based plastic design
NGM: Normalized ground motion
NLSPA: Non linear static pushover analysis
NLTHA: Non linear time history analysis
PBD: Performance based design
PBPD: Performance based plastic design
PGA: Peak ground acceleration
PGA_max_: Maximum peak ground acceleration
PGV: Peak ground velocity
PGV_max_: Maximum peak ground velocity
RC-SMRF: Reinforced concrete special moment resisting Frames

## Notations

${A}_{h}$: Coefficient of horizontal acceleration.
$${d}_{b}$$: Bar diameter
${f}_{c,exp}$: Compressive strength of concrete expected
${f}_{ck}$: Characteristic compressive strength
${f}_{y,exp}$: Yield strength of steel expected
$${f}_{y}$$: Yield strength
$$g$$: Gravitational acceleration
${h}_{i}$: I^th^ level frame height
${h}_{n}$: Frame height at top
*H1*: Seismic acceleration in 1 horizontal direction
*H2*: Seismic acceleration in 2 horizontal direction
$$I$$: Importance factor
${K}_{e}$: Elastic/ Effective stiffness
${K}_{i}$: Post yield stiffness
${L}_{P}$: Plastic hinge length
$$M$$: Total mass
${Q}_{i}$: I^th^ level storey shear
$R$: Response Reduction factor
${R}_{\mu }$: Strength reduction factor
$${S}_{a}/g$$: Spectral acceleration
${S}_{V}$: Spectral velocity of system
$$T$$: Fundamental period
${V}_{B}$: Total base shear
${V}_{B}/W$: Base shear ratio
${V}_{y}$: Yield shear strength
${V}_{e}$: Elastic shear strength
${V}_{max}$: Peak base shear
$W$: Lumped seismic weight
${W}_{e}$: Total elastic energy
${W}_{p}$: Total Plastic energy
${W}_{i}$: I^th^ level lumped weight at
${W}_{n}$: Top storey Lumped weight
$\Upsilon$: Energy modification factor
${\Upsilon }_{o}$: Modified energy modification factor
$$\mu$$: Ductility factor
$${\delta }_{max}$$: Maximum displacement
$${\delta }_{e}$$: Elastic displacement
$${\delta }_{y}$$: Yield displacement
${Rt}_{o}$: Post yield stiffness ratio
$${\mu }_{o}$$: Standard mean
${\theta }_{max}$: Target drift selected
${\theta }_{y}$: Yield drifts selected
$${\theta }_{p}$$: Plastic drift
β: PBPD Shear distribution factor
${\sigma }_{c}$: Standard deviation of concrete
${\sigma }_{s}$: Standard deviation of steel
${\upbeta }_{o}$: Generalized Standard deviation
