# Supplementary material for: A comparative study of modified performance based plastic design methods for seismic design of RC frames: improvement over existing methods
Source: Sci Rep. 2026 Apr 4;16:16239. doi: 10.1038/s41598-026-45796-4 (PMC13201680; doi:10.1038/s41598-026-45796-4)
Supplement: Supplementary file 1 — Supplementary Information. [file 41598_2026_45796_MOESM1_ESM.docx]

**Supplementary Materials**

**Table a.** Section details of 6 storey FBD frame

| S  T  O  R  E  Y | Beam Sections | | | Interior Column Sections | | Exterior Column Sections | |
| --- | --- | --- | --- | --- | --- | --- | --- |
|  | Size (mm) | Bottom  Steel pt. % | Top  Steel  pt. % | Size (mm) | Steel  p_t._% | Size (mm) | Steel  p_t._% |
| 6 | 250 X 380 | 0.75 | 0.75 | 350 X 350 | 1.31 | 350 X 350 | 1.42 |
| 5 | 250 X 380 | 0.75 | 0.96 | 350 X 350 | 1.31 | 350 X 350 | 1.42 |
| 4 | 250 X 380 | 0.75 | 1.2 | 350 X 350 | 1.31 | 350 X 350 | 1.42 |
| 3 | 250 X 400 | 1.14 | 1.64 | 400 X 400 | 1.12 | 400 X 400 | 1.01 |
| 2 | 250 X 400 | 1.23 | 1.74 | 400 X 400 | 1.12 | 400 X 400 | 1.01 |
| 1 | 250 X 400 | 1.20 | 1.67 | 400 X 400 | 1.12 | 400 X 400 | 1.01 |

**Table b.** Section details of 6 storey PBPD frame

| S  T  O  R  E  Y | Beam Sections | | | Interior Column Sections | | Exterior Column Sections | |
| --- | --- | --- | --- | --- | --- | --- | --- |
|  | Size (mm) | Bottom  Steel pt. % | Top  Steel  pt. % | Size (mm) | Steel  p_t._% | Size (mm) | Steel  p_t._% |
| 6 | 230 X 350 | 0.78 | 1.28 | 350 X 350 | 3.19 | 350 X 350 | 2.99 |
| 5 | 230 X 350 | 0.78 | 1.28 | 350 X 350 | 3.19 | 350 X 350 | 2.99 |
| 4 | 230 X 350 | 0.78 | 1.28 | 350 X 350 | 3.19 | 350 X 350 | 2.99 |
| 3 | 250 X 380 | 0.87 | 1.30 | 430 X 430 | 3.08 | 430 X 430 | 2.95 |
| 2 | 250 X 380 | 0.87 | 1.30 | 430 X 430 | 3.08 | 430 X 430 | 2.95 |
| 1 | 250 X 380 | 0.87 | 1.30 | 430 X 430 | 3.08 | 430 X 430 | 2.95 |

**Table c.** Section details of 6 storey MPBPD frame

| S  T  O  R  E  Y | Beam Sections | | | Interior Column Sections | | Exterior Column Sections | |
| --- | --- | --- | --- | --- | --- | --- | --- |
|  | Size (mm) | Bottom  Steel pt. % | Top  Steel  pt. % | Size (mm) | Steel  p_t._% | Size (mm) | Steel  p_t._% |
| 6 | 250 X 380 | 1.02 | 1.18 | 350 X 350 | 1.68 | 350 X 350 | 1.68 |
| 5 | 250 X 380 | 1.02 | 1.18 | 350 X 350 | 1.68 | 350 X 350 | 1.68 |
| 4 | 250 X 400 | 0.96 | 1.10 | 400 X 400 | 1.32 | 400 X 400 | 1.29 |
| 3 | 250 X 400 | 0.96 | 1.10 | 400 X 400 | 1.32 | 400 X 400 | 1.29 |
| 2 | 250 X 430 | 0.98 | 1.12 | 450 X 450 | 1.50 | 450 X 450 | 1.42 |
| 1 | 250 X 430 | 0.98 | 1.12 | 450 X 450 | 1.50 | 450 X 450 | 1.42 |

**Table d.** Section details of 12 storey FBD frame

| S  T  O  R  E  Y | Beam Sections | | | Interior Column Sections | | Exterior Column Sections | |
| --- | --- | --- | --- | --- | --- | --- | --- |
|  | Size (mm) | Bottom  Steel pt. % | Top  Steel  pt. % | Size (mm) | Steel  p_t._% | Size (mm) | Steel  p_t._% |
| 12 | 250 X 400 | 0.61 | 0.61 | 500 X 500 | 1.94 | 500 X 500 | 2.01 |
| 11 | 250 X 400 | 0.61 | 0.98 | 500 X 500 | 1.94 | 500 X 500 | 2.01 |
| 10 | 250 X 400 | 0.68 | 1.08 | 500 X 500 | 1.94 | 500 X 500 | 2.01 |
| 9 | 250 X 400 | 0.77 | 1.34 | 500 X 500 | 1.94 | 500 X 500 | 2.01 |
| 8 | 300 X 450 | 0.69 | 1.21 | 600 X 600 | 1.36 | 600 X 600 | 1.40 |
| 7 | 300 X 450 | 0.69 | 1.15 | 600 X 600 | 1.36 | 600 X 600 | 1.40 |
| 6 | 300 X 450 | 0.71 | 1.19 | 600 X 600 | 1.36 | 600 X 600 | 1.40 |
| 5 | 300 X 450 | 0.73 | 1.07 | 600 X 600 | 1.36 | 600 X 600 | 1.40 |
| 4 | 300 X 500 | 0.73 | 1.09 | 800 X 800 | 0.92 | 800 X 800 | 0.98 |
| 3 | 300 X 500 | 0.71 | 1.07 | 800 X 800 | 0.92 | 800 X 800 | 0.98 |
| 2 | 300 X 500 | 0.69 | 0.99 | 800 X 800 | 0.92 | 800 X 800 | 0.98 |
| 1 | 300 X 500 | 0.63 | 0.79 | 800 X 800 | 0.92 | 800 X 800 | 0.98 |

**Table e.** Section details of 12 storey PBPD frame

| S  T  O  R  E  Y | Beam Sections | | | Interior Column Sections | | Exterior Column Sections | |
| --- | --- | --- | --- | --- | --- | --- | --- |
|  | Size (mm) | Bottom  Steel pt. % | Top  Steel  pt. % | Size (mm) | Steel  p_t._% | Size (mm) | Steel  p_t._% |
| 12 | 230 X 350 | 0.72 | 0.72 | 500 X 500 | 1.82 | 500 X 500 | 1.78 |
| 11 | 230 X 350 | 0.72 | 0.72 | 500 X 500 | 1.82 | 500 X 500 | 1.78 |
| 10 | 230 X 350 | 0.72 | 0.72 | 500 X 500 | 1.82 | 500 X 500 | 1.78 |
| 9 | 230 X 350 | 0.72 | 0.72 | 500 X 500 | 1.82 | 500 X 500 | 1.78 |
| 8 | 250 X 400 | 0.81 | 0.88 | 600 X 600 | 1.69 | 600 X 600 | 1.58 |
| 7 | 250 X 400 | 0.81 | 0.88 | 600 X 600 | 1.69 | 600 X 600 | 1.58 |
| 6 | 250 X 400 | 0.81 | 0.88 | 600 X 600 | 1.69 | 600 X 600 | 1.58 |
| 5 | 250 X 400 | 0.81 | 0.88 | 600 X 600 | 1.69 | 600 X 600 | 1.58 |
| 4 | 300 X 450 | 0.98 | 1.09 | 850 X 850 | 0.84 | 850 X 850 | 0.80 |
| 3 | 300 X 450 | 0.98 | 1.09 | 850 X 850 | 0.84 | 850 X 850 | 0.80 |
| 2 | 300 X 450 | 0.98 | 1.09 | 850 X 850 | 0.84 | 850 X 850 | 0.80 |
| 1 | 300 X 450 | 0.98 | 1.09 | 850 X 850 | 0.84 | 850 X 850 | 0.80 |

**Table f.** Section details of 12 storey MPBPD frame

| S  T  O  R  E  Y | Beam Sections | | | Interior Column Sections | | Exterior Column Sections | |
| --- | --- | --- | --- | --- | --- | --- | --- |
|  | Size (mm) | Bottom  Steel pt. % | Top  Steel  pt. % | Size (mm) | Steel  p_t._% | Size (mm) | Steel  p_t._% |
| 12 | 250 X 400 | 0.6 | 1.02 | 500 X 500 | 2.36 | 500 X 500 | 2.20 |
| 11 | 250 X 400 | 0.6 | 1.02 | 500 X 500 | 2.36 | 500 X 500 | 2.20 |
| 10 | 250 X 400 | 0.78 | 1.36 | 500 X 500 | 2.36 | 500 X 500 | 2.20 |
| 9 | 250 X 400 | 0.78 | 1.36 | 500 X 500 | 2.36 | 500 X 500 | 2.20 |
| 8 | 300 X 450 | 0.75 | 1.23 | 650 X 650 | 1.86 | 650 X 650 | 1.78 |
| 7 | 300 X 450 | 0.75 | 1.23 | 650 X 650 | 1.86 | 650 X 650 | 1.78 |
| 6 | 300 X 450 | 0.75 | 1.23 | 650 X 650 | 1.86 | 650 X 650 | 1.78 |
| 5 | 300 X 450 | 0.75 | 1.23 | 650 X 650 | 1.86 | 650 X 650 | 1.78 |
| 4 | 300 X 500 | 0.75 | 1.09 | 850 X 850 | 1.09 | 850 X 850 | 0.98 |
| 3 | 300 X 500 | 0.75 | 1.09 | 850 X 850 | 1.09 | 850 X 850 | 0.98 |
| 2 | 300 X 500 | 0.75 | 1.09 | 850 X 850 | 1.09 | 850 X 850 | 0.98 |
| 1 | 300 X 500 | 0.75 | 1.09 | 850 X 850 | 1.09 | 850 X 850 | 0.98 |
